# Resveratrol Ameliorates High-Fat-Diet-Induced Abnormalities in Hepatic Glucose Metabolism in Mice via the AMP-Activated Protein Kinase Pathway

**DOI:** 10.1155/2021/6616906

**Published:** 2021-06-25

**Authors:** Caiping Lu, Hanying Xing, Linquan Yang, Kaiting Chen, Linyi Shu, Xiaojian Zhao, Guangyao Song

**Affiliations:** ^1^Department of Internal Medicine, Hebei Medical University, Shijiazhuang 050017, Hebei Province, China; ^2^Department of Endocrinology, Hebei General Hospital, Shijiazhuang 050051, Hebei Province, China; ^3^Department of Endocrinology, Shijiazhuang People's Hospital, Shijiazhuang 050000, Hebei Province, China; ^4^Hebei Key Laboratory of Metabolic Diseases, Hebei General Hospital, Shijiazhuang 050051, Hebei Province, China; ^5^Department of Thoracic Surgery, Shijiazhuang People's Hospital, Shijiazhuang 050000, Hebei Province, China

## Abstract

Diabetes mellitus is highly prevalent worldwide. High-fat-diet (HFD) consumption can lead to liver fat accumulation, impair hepatic glycometabolism, and cause insulin resistance and the development of diabetes. Resveratrol has been shown to improve the blood glucose concentration of diabetic mice, but its effect on the abnormal hepatic glycometabolism induced by HFD-feeding and the mechanism involved are unknown. In this study, we determined the effects of resveratrol on the insulin resistance of high-fat-diet-fed mice and a hepatocyte model by measuring serum biochemical indexes, key indicators of glycometabolism, glucose uptake, and glycogen synthesis in hepatocytes. We found that resveratrol treatment significantly ameliorated the HFD-induced abnormalities in glucose metabolism in mice, increased glucose absorption and glycogen synthesis, downregulated protein phosphatase 2A (PP2A) and activated Ca^2+^/CaM-dependent protein kinase kinase *β* (CaMKK*β*), and increased the phosphorylation of AMP-activated protein kinase (AMPK). In insulin-resistant HepG2 cells, the administration of a PP2A activator or CaMKK*β* inhibitor attenuated the effects of resveratrol, but the administration of an AMPK inhibitor abolished the effects of resveratrol. Resveratrol significantly ameliorates abnormalities in glycometabolism induced by HFD-feeding and increases glucose uptake and glycogen synthesis in hepatocytes. These effects are mediated through the activation of AMPK by PP2A and CaMKK*β*.

## 1. Introduction

Diabetes mellitus (DM) is characterized by the dysregulation of carbohydrate, protein, and fat metabolism, which is caused by insufficient insulin secretion or insulin resistance (IR). It is a chronic endocrine system disorder that principally manifests as chronic hyperglycemia [1]. Most cases of diabetes can be classified as type 1 or type 2 diabetes mellitus (T2DM), with T2DM accounting for 90%–95% of all cases. T2DM is associated with chronic organ damage, dysfunction, and failure and therefore is associated with a large financial burden worldwide. The disease involves both IR and defects in pancreatic *β*-cell function and mass and is frequently linked to obesity [2]. The recent increase in the prevalence of T2DM has largely been the result of increases in the consumption of high-energy diets, a sedentary lifestyle, and obesity [3].

IR is the best predictor of the development of diabetes and is a consistent finding in patients with T2DM. The chronic consumption of a high-fat diet (HFD) can result in an abnormal accumulation of fat in the liver, which has toxic effects that contribute to the pathogenesis of T2DM and the related metabolic syndrome (MetS) [4, 5]. The cellular energy sensor AMP-activated protein kinase (AMPK) is known to be a key mediator of glucose metabolism and is regarded as a potential therapeutic target for diabetes and its complications.

Resveratrol (3, 5, 4'‐trihydroxystilbene) is a polyphenol plant‐derived compound. It is found at high concentrations in grapes, berries, and peanuts and is commercially available in the form of dietary supplements. Previous studies have shown that resveratrol has a wide range of biological effects, including antimicrobial, anti-inflammatory, antiapoptotic, and anticancer effects [6, 7], and it promotes the formation of osteogenic factors [8]. It also has antioxidant effects in certain chronic diseases, such as diabetes and cardiovascular disease. Previous studies conducted in humans and animals have shown that resveratrol reduces blood glucose by having effects on insulin secretion and glucose uptake [9]. Protein phosphatase 2A (PP2A) and Ca^2+^/CaM-dependent protein kinase kinase *β* (CaMKK*β*) are important regulator of AMPK [10]. PP2A and CaMKK*β* play important roles in the regulation of glycometabolism in hepatocytes [11, 12], but whether resveratrol influences glycometabolism in hepatocytes and whether these molecules are involved is unclear. Therefore, we aimed to use animal and cell models to clarify the effects of resveratrol on hepatic glucose metabolism and the molecular mechanisms involved.

## 2. Materials and Methods

### 2.1. Animals and Groups

Six-to-eight-week-old male C57BL/6J mice were housed in individual cages under controlled temperature (23°C) and illumination (12-hour light/dark cycle) conditions. Model mice were fed an HFD (60% fat, 20% protein, and 20% carbohydrate, Research Diets) for 16 weeks, and each mouse was given 4 g daily. The mice in the resveratrol intervention group were given the HFD and given resveratrol 60, 80, and 100 mg/kg body weight/day by gavage from 4 to 16 weeks. At the end of the study, the mice were killed by decapitation. All the experiments and procedures involved in this study were approved by the Ethics Committee of the USC and were performed in accordance with EU legislation regarding the use of experimental animals.

### 2.2. Materials

Triglyceride (TG), total cholesterol (TC), and low-density lipoprotein-cholesterol (LDL-c) were from Roche Life Science (Basel, Switzerland). 2-Deoxy-2-[(7-nitro-2,1,3-benzoxadiazol-4-yl)amino]-D-glucose (2-NBDG), STO-609 (CaMKK*β* inhibitor), compound C (AMPK inhibitor), and DT-061 (PP2A activator) were from Sigma-Aldrich (St. Louis, United States), and PP2A, AMPK, p-AMPK, CaMKK*β*, p- CaMKK*β,* and GAPDH were purchased from Abcam (Cambridge, UK).

### 2.3. Biochemical Analyses

Serum triglyceride (TG), total cholesterol (TC), low-density lipoprotein-cholesterol (LDL-c), and high-density lipoprotein-cholesterol (HDL-c) concentrations were measured using an automatic biochemical analyzer (200FR, Toshiba, Japan).

### 2.4. Assessment of Insulin Resistance

The homeostasis model assessment of insulin resistance (HOMA-IR) was calculated as follows: HOMA-IR = (fasting insulin concentration (*µ*U/ml)) × (fasting glucose concentration (mmol/l))/22.5.

### 2.5. Intraperitoneal Glucose Tolerance Testing

After the final dose of resveratrol was administered, the mice were fasted for 12 h and then intraperitoneally injected with 2.0 g/kg glucose (50% glucose: 0.9% sodium chloride, 1 : 1). Blood samples were then collected from a tail vein 0, 15, 30, 60, and 120 min later. The area under the blood glucose curve (AUC) was calculated as follows (Dong et al., 2014): AUC = (basal glycemia + glycemia at 0.25 h) × 0.25 + (glycemia at 0.5 h + glycemia at 0.25 h) × 0.25 + (glycemia at 1 h + glycemia at 0.5 h) × 0.25 + (glycemia at 1 h + glycemia at 2 h) × 0.25.

### 2.6. HepG2 Cell Culture and the Induction of Insulin Resistance

HepG2 cells were obtained from the Peking Union Medical College and maintained in Dulbecco's-modified Eagle's medium (DMEM) containing 25 mM glucose, 10% FBS, penicillin (100 units/ml), and streptomycin (100 *µ*g/ml) at 37°C in a humidified atmosphere of 95% air and 5% CO_2_. To induce insulin resistance, HepG2 cells were incubated with 0.25 mM palmitate (PA) for 24 h in serum-free medium, followed by incubation in medium containing 20 nM irisin or vehicle (PBS) for 30 min for measurement of protein phosphorylation, or 24 h for other measurements, in the presence of resveratrol. To explore the role of the AMPK signaling pathways in resveratrol-stimulated 2-deoxy-2-[(7-nitro-2,1,3-benzoxadiazol-4-yl)amino]-D-glucose (2-NBDG) uptake, HepG2 cells were incubated with resveratrol for 24 h in the presence or absence of 27 *µ*M STO-609 (CaMKK*β* inhibitor), 20 *µ*M compound C (AMPK inhibitor), or 30 *µ*M of DT-061 (PP2A activator) for 30 min. Subsequently, the cells were used for measurements including glucose uptake assay.

### 2.7. Glucose Uptake and Glycogen Synthesis

Glucose uptake was measured after the addition of the tracer 2-NBDG to the culture medium, as previously reported. The accumulation of glycogen was determined using a Glycogen Colorimetric/Fluorometric Assay Kit (K646-100, BioVision, USA), as described previously.

### 2.8. Western Blot Analysis

Briefly, liver samples (100 mg) were minced and homogenized in 1 mL ice-cold RIPA buffer supplemented with a complete protease inhibitor cocktail. After centrifugation at 12,000xg for 5 min at 4°C, the protein concentrations of the samples were determined using a bicinchoninic acid protein assay. A total of 30 *µ*g of denatured protein per sample was separated by 10% sodium dodecyl sulfate-polyacrylamide gel electrophoresis and transferred to PVDF membranes. After being blocked with 5% nonfat milk in Tris-buffered saline containing 0.1% Tween 20 (TBST) for 1.5 h at room temperature, the membranes were incubated overnight at 4°C with a primary antibody (1 : 1,000). The membranes were then washed with TBST and incubated with horseradish peroxidase-conjugated secondary antibodies (1 : 3,000) for 1 h at room temperature. Immunoreactive protein bands were visualized using an enhanced chemiluminescence reagent kit and their intensities were calculated using Quantity One software. Glyceraldehyde 3-phosphate dehydrogenase (GAPDH) was used as the loading control.

### 2.9. Statistical Analyses

Statistical analyses were performed using SPSS v.17.0 software (IBM, Inc., Armonk, NY, USA). All data are shown as means ± standard deviations (SDs). One-way analysis of variance (ANOVA) was used for multiple group comparisons, followed by least significant difference tests for comparisons of two groups, as appropriate. *P* < 0.05 was considered to indicate statistical significance. All experiments were performed in triplicate and repeated at least three times.

## 3. Results

### 3.1. Resveratrol Ameliorates Abnormalities in Lipid and Glucose Metabolism in HFD-Fed Mice

**Figure 1:**
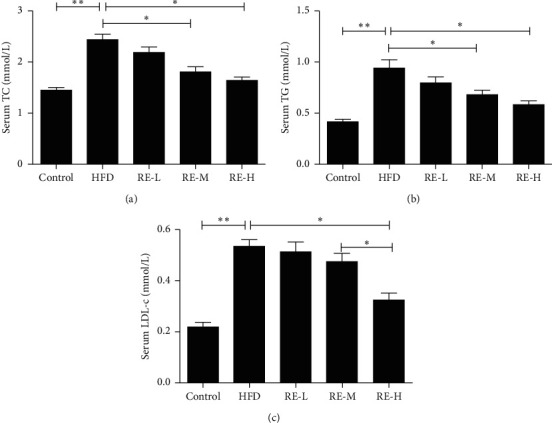
Resveratrol improves lipid homeostasis in HFD-induced mice. Effect of resveratrol on TC, TG, and LDL-c. Pretreatment with resveratrol, RE-L (60 mg/kg), RE-M (80 mg/kg), and RE-H (100 mg/kg). The data shown represent the means ± SEM. ^*∗*^*P* < 0.05; ^*∗∗*^*P* < 0.01.

**Figure 2:**
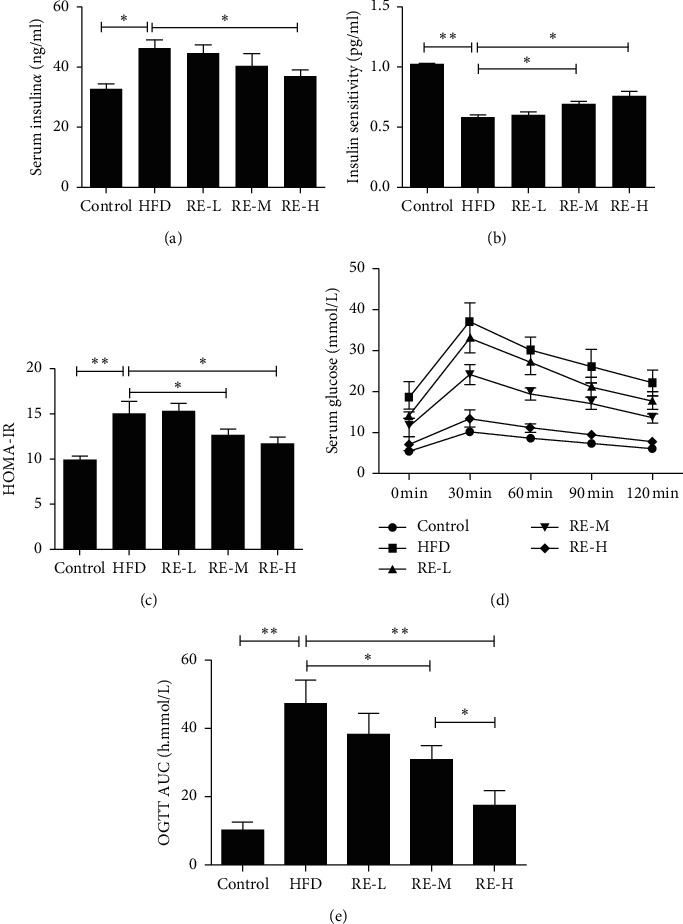
Resveratrol improves serum glucose and protects against HFD-induced insulin resistance. Serum levels of blood glucose, insulin, insulin sensitivity, and OGTT were measured after 8 weeks of treatment. The insulin sensitivity index (ISI) was measured according to the formula ISI = 1/(fasting insulin × fasting plasma glucose). The HOMA-IR index of IR was determined as follows: blood glucose (mmol/L) × serum insulin (mg/ml)/22.5. Pretreatment with resveratrol, RE-L (60 mg/kg), RE-M (80 mg/kg), and RE-H (100 mg/kg). The data shown represent the means ± SEM. ^*∗*^*P* < 0.05, ^*∗∗*^*P* < 0.01.

HFD consumption significantly increased the serum concentrations of TC and TG in the mice (the serum TC and TG concentrations of high-fat-diet-fed mice were 2.42 mmol/L and 0.84 mmol/L, and those of control mice were 1.57 mmol/L and 0.41 mmol/L, respectively). However, the TC and TG concentrations in mice preadministered resveratrol were significantly lower than in HFD model mice. In particular, there were significant differences between the moderate- and high-dose groups and the HFD model group (*P* < 0.05, Figure 1). HFD-feeding increased the serum LDL-c concentration and reduced the serum HDL-c concentration in HFD model mice, but the preadministration of resveratrol ameliorated these effects (*P* < 0.05, Figure 1). HFD-feeding also significantly increased the blood glucose and serum insulin concentrations, reduced the insulin sensitivity, and increased the HOMA-IR score of the mice. However, resveratrol administration significantly ameliorated the abnormal blood glucose, serum insulin, and insulin sensitivity of the mice (*P* < 0.05, Figure 2). Glucose tolerance testing showed that there were significant differences between HFD model mice and control mice, and high-dose resveratrol group and the HFD model group also had significant difference (*P* < 0.05, Figure 2).

### 3.2. Resveratrol Increases Glucose Uptake and Glycogen Synthesis in HepG2 Cells

**Figure 3:**
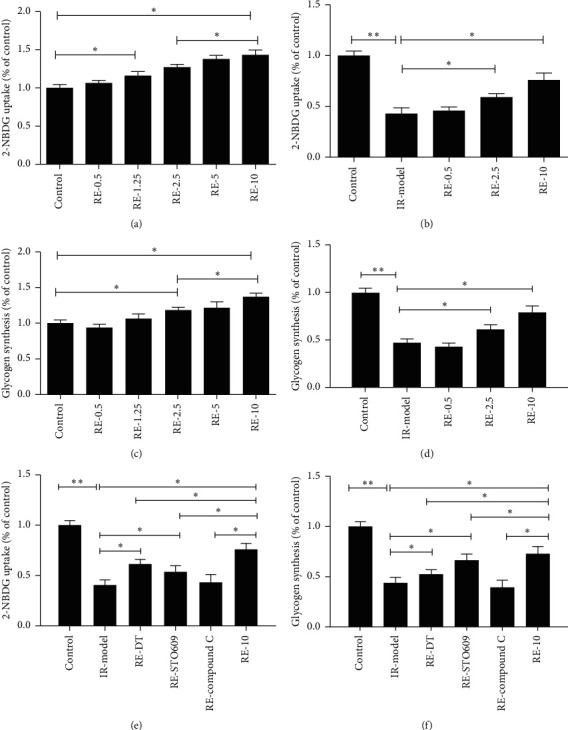
Effects of different doses of resveratrol on 2-NBDG uptake and glycogen synthesis in HepG2 cells. Dose-dependent effect of resveratrol on 2-NBDG uptake in the IR-model and glycogen synthesis in the IR-model. The effect of resveratrol combined with different inhibitors on 2-NBDG uptake and glycogen synthesis. All experiments were performed at least 3 times. ^*∗*^*P* < 0.05; ^*∗∗*^*P* < 0.01.

Glucose uptake by HepG2 cells was measured using 2-NBDG as a tracer. Resveratrol treatment dose and time dependently increased the uptake of 2-NBDG (*P* < 0.05, Figure 3). Resveratrol also significantly increased glycogen synthesis in the hepatocytes (*P* < 0.05, Figure 3). At the same time, cells were stimulated with palmitate (PA) to establish insulin resistance cell model. After resveratrol was given, the changes of cell glucose uptake and glycogen synthesis in insulin resistance hepG2 cell were detected. The results showed that resveratrol could also significantly increase 2-NBDG uptake and glycogen synthesis in insulin resistant hepatocyte model (*P* < 0.05, Figure 3).

### 3.3. The Effects of Resveratrol on Hepatocyte Glycometabolism Are Mediated through the Activation of AMPK, via the PP2A and CaMKK*β* Pathways

**Figure 4:**
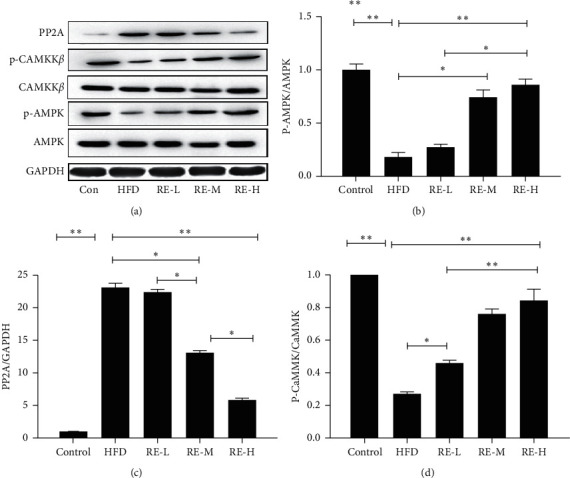
Effect of resveratrol on PP2A AMPK, and CaMKK*β* in HFD amidated mice. Western blots for PP2A, AMPK, and CaMKK*β*. All experiments were performed at least 3 times. ^*∗*^*P* < 0.05; ^*∗∗*^*P* < 0.01.

**Figure 5:**
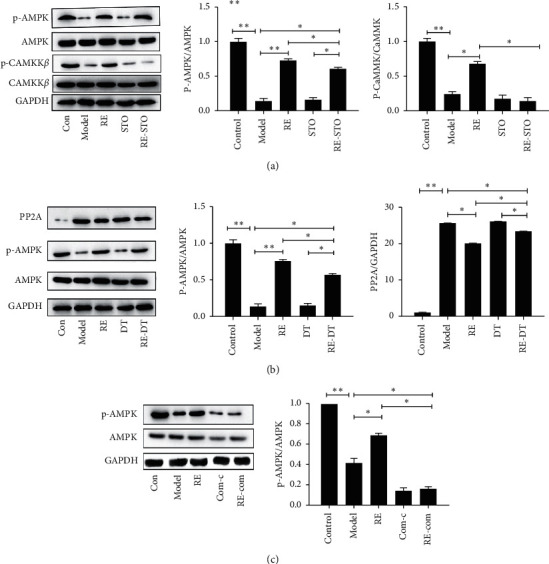
Effect of resveratrol on PP2A, AMPK, and CaMKK*β* in HepG2 cells. Western blots for PP2A, AMPK, and CaMKK*β* for resveratrol combined with STO-609 (CaMKK*β* inhibitor) (a), DT-061 (PP2A activator) (b), or compound C (AMPK inhibitor) (c). All experiments were performed at least 3 times. ^*∗*^*P* < 0.05; ^*∗∗*^*P* < 0.01.

Western blot analysis showed that resveratrol significantly increased the concentrations of p-CaMKK*β*, and p-AMPK in the livers of HFD-fed mice decreased the PP2A level (*P* < 0.05, Figure 4). To further clarify the mechanism whereby resveratrol increases glucose uptake and glycogen synthesis in hepatocytes, cells were coincubated with PP2A agonists, CaMKK*β* inhibitor, and resveratrol. This caused a significant reduction in the effects of resveratrol on glucose uptake and glycogen synthesis *versus* resveratrol-treated cells, but both remained significantly higher than in untreated insulin-resistant cells. However, when cells were coincubated with resveratrol and an AMPK inhibitor, the effects of resveratrol on glucose uptake and glycogen synthesis were abolished, such that there was no significant difference in either from the untreated insulin-resistant cells (*P* < 0.05, Figure 5). The results of showed that coincubation of cells with resveratrol and PP2A agonists and CaMKK*β* inhibitors significantly reduced the effect of AMPK, but significant differences *versus* the insulin-resistant cells remained. However, coincubation with an AMPK inhibitor abolished the effects of resveratrol.

## 4. Discussion

Obesity is one of the most significant current health issues worldwide. In 2018, 800 million adults (13% of the world's population) were classified as obese. Obesity increases the risk of developing several diseases, including T2DM, MetS, various cancers, cardiovascular disease, and cognitive defects, such as Alzheimer's disease [13–15]. T2DM is a metabolic disease that is characterized by chronic hyperglycemia and can be accompanied by complications, including of the cardiovascular system. As the prevalence of T2DM rate has gradually increased, strategies to prevent and treat T2DM have become important foci of global research. Modern environmental factors, such as a western-style dietary pattern (energy-dense foods, rich in fat) and a sedentary lifestyle, largely explain the rapid increase in the number of patients with diabetes. HFD consumption can lead to IR, high blood glucose, liver fat accumulation, changes in the intestinal microbiota, and even cognitive impairment [16, 17]. In the present study, resveratrol treatment significantly ameliorated the increases in serum TC and TG concentrations that were induced by HFD-feeding in mice. The effects of resveratrol were dose dependent, with significant differences between the moderate- and high-dose groups and the HFD model group. HFD feeding also increased the circulating concentrations of glucose and LDL-c in the mice, but these effects were significantly ameliorated by pretreatment with resveratrol. Finally, HFD-feeding induced insulin resistance and abnormal glucose metabolism, as shown by the high serum insulin concentration and HOMA-IR, but this insulin resistance was significantly ameliorated by resveratrol treatment.

T2DM can be managed through dietary modification, regular exercise, weight control, and patient education, such that the use of pharmacological therapy can be avoided. Several previous studies have shown that the pharmacological activation of AMPK in insulin‐resistant rodents improves their serum lipid profile, blood glucose homeostasis, and blood pressure [13, 18, 19]. Therefore, AMPK may represent a therapeutic target for the management of T2DM. Some study showed that resveratrol enhanced brown adipocyte formation and thermogenic function in mouse iBAT by promoting the expression of brown adipogenic markers via activating AMPK*α*1 [20]. Resveratrol can also activate the AMPK pathway in kidney tissue and then improve the occurrence of renal injury caused by diabetes [21]. At the same time, through the study of AMPK knockout mice, resveratrol significantly reduced the improvement of diabetes in AMPK deficient mice [22]. However, how resveratrol activates AMPK and then plays a role and whether resveratrol affects the synthesis of glucose by affecting AMPK pathway in hepatocytes remains to be clarified. The liver is the key organ of glucose and lipid metabolism. The present results show that resveratrol activates liver AMPK (upregulates p-AMPK), but the mechanism involved remains to be elucidated. To define the molecular mechanism underlying resveratrol action, we aimed to identify the signaling pathways that were involved in the stimulation of 2-NBDG uptake into HepG2 cells by resveratrol.

We found that resveratrol significantly increased 2-NBDG uptake in a HepG2 cell model of palmitate-induced insulin resistance (Figure 2). Liver glycogen synthesis is an important component of whole-body glucose homeostasis and serum is upregulated by insulin. Here, we have shown that resveratrol significantly increases glycogen synthesis in hepatocytes. Previous studies have shown that the PP2A pathways are involved in the regulation of glycometabolism in hepatocytes, but it was unknown whether these pathways are involved in the mechanism of action of resveratrol. Therefore, we coincubated HepG2 cells with resveratrol and PP2A agonists, and results found that when resveratrol was added alongside a PP2A agonists, the effect of resveratrol on hepatocyte glycometabolism was significantly reduced, but the significant difference from the insulin resistant cells remained. However, coincubation with an AMPK inhibitor abolished the effects of resveratrol on hepatocyte glycometabolism, such that there was no significant difference between the untreated insulin-resistant cells and resveratrol-treated cells. These results imply that the effects of resveratrol on glycometabolism in hepatocytes are mediated through the AMPK pathway. Activation of AMPK can phosphorylate its active site through inhibited PP2A. CaMKK*β* is another key regulator of AMPK [23, 24]. To further elucidate the mechanism whereby resveratrol activates AMPK, we coincubated HepG2 cells with a CaMKK*β* inhibitor and resveratrol. This showed that although inhibition of CaMKK*β* alone did not inhibit the activation of AMPK by resveratrol, both the resveratrol-induced activation of AMPK and its effects on glycometabolism in hepatocytes could be largely prevented by PP2A and CaMKK*β*.

## 5. Conclusions

In conclusion, resveratrol significantly ameliorates the insulin resistance and abnormal glucose metabolism of HFD-fed mice and activates AMPK pathways in the livers of these mice. The molecular mechanism of the effects of resveratrol on glycometabolism in hepatocytes involves the regulated PP2A and CaMKK*β* and the consequent activation of AMPK.

## Data Availability

The data used to support the findings of this study are available from the corresponding author upon request.

## References

[1] Khandelwal S. (2020). Obesity in midlife: lifestyle and dietary strategies. *Climacteric*.

[2] Popa-Wagner A., Dumbrava D.-A., Dumitrascu D. (2020). Dietary habits, lifestyle factors and neurodegenerative diseases. *Neural Regeneration Research*.

[3] Browning J. D., Szczepaniak L. S., Dobbins R. (2004). Prevalence of hepatic steatosis in an urban population in the United States: impact of ethnicity. *Hepatology*.

[4] Bae S.-A., Fang M. Z., Rustgi V., Zarbl H., Androulakis I. P. (2019). At the interface of lifestyle, behavior, and circadian rhythms: metabolic implications. *Frontiers in Nutrition*.

[5] Hodson L., Gunn P. J. (2019). The regulation of hepatic fatty acid synthesis and partitioning: the effect of nutritional state. *Nature Reviews Endocrinology*.

[6] Ginsberg H. N., Elam M. B., Lovato L. C. (2010). Effects of combination lipid therapy in type 2 diabetes mellitus. *New England Journal of Medicine*.

[7] Chen J., Cao X., Cui Y., Zeng G., Chen J., Zhang G. (2018). Resveratrol alleviates lysophosphatidylcholine-induced damage and inflammation in vascular endothelial cells. *Molecular Medicine Reports*.

[8] Seo C. H., Kim J.-B. (2015). Therapeutic potential of resveratrol in type I Gaucher disease. *Phytotherapy Research*.

[9] Cheang W. S., Wong W. T., Wang L. (2019). Resveratrol ameliorates endothelial dysfunction in diabetic and obese mice through sirtuin 1 and peroxisome proliferator-activated receptor *δ*. *Pharmacological Research*.

[10] Scicali R., Di Pino A., Ferrara V. (2018). New treatment options for lipid-lowering therapy in subjects with type 2 diabetes. *Acta Diabetologica*.

[11] Lee M. Y., Chen W. C., Hsu W. H., Chen S. C. (2019). Lee JC liraglutide inhibits hepatitis C virus replication through an AMP activated protein kinase dependent mechanism. *International Journal of Molecular Sciences*.

[12] He W. Y., Zhang B., Zhao W. C (2019). mTOR activation due to APPL1 deficiency exacerbates hyperalgesia via Rab5/Akt and AMPK signaling pathway in STZ-induced diabetic rats. *Molecular Pain*.

[13] Nguyen-Ngo C., Jayabalan N., Salomon C., Lappas M. (2019). Molecular pathways disrupted by gestational diabetes mellitus. *Journal of Molecular Endocrinology*.

[14] Liu J., Wang Y., Lin L. (2019). Small molecules for fat combustion: targeting obesity. *Acta Pharmaceutica Sinica B*.

[15] Joshi T., Singh A. K., Haratipour P. (2019). Targeting AMPK signaling pathway by natural products for treatment of diabetes mellitus and its complications. *Journal of Cellular Physiology*.

[16] Zhang J., Chen Y., Luo H. (2018). Recent update on the pharmacological effects and mechanisms of dihydromyricetin. *Frontiers in Pharmacology*.

[17] Alnahdi A., John A., Raza H. (2019). Augmentation of glucotoxicity, oxidative stress, apoptosis and mitochondrial dysfunction in HepG2 cells by palmitic acid. *Nutrients*.

[18] Liao Z., Zhang J., Wang J. (2019). The anti-nephritic activity of a polysaccharide from okra (*Abelmoschus esculentus* (L.) Moench) via modulation of AMPK-Sirt1-PGC-1*α* signaling axis mediated anti-oxidative in type 2 diabetes model mice. *International Journal of Biological Macromolecules*.

[19] Zhou J. Y., Poudel A., Welchko R. (2019). Liraglutide improves insulin sensitivity in high fat diet induced diabetic mice through multiple pathways. *European Journal of Pharmacology*.

[20] Wang S., Liang X., Yang Q. (2017). Resveratrol enhances brown adipocyte formation and function by activating AMP-activated protein kinase (AMPK) *α*1 in mice fed high-fat diet. *Molecular Nutrition & Food Research*.

[21] Guo H., Zhang L. (2018). Resveratrol provides benefits in mice with type II diabetes-induced chronic renal failure through AMPK signaling pathway. *Experimental and Therapeutic Medicine*.

[22] Um J.-H., Park S.-J., Kang H. (2010). AMP-activated protein kinase-deficient mice are resistant to the metabolic effects of resveratrol. *Diabetes*.

[23] Lim J. H., Kim H. W., Kim M. Y. (2018). Cinacalcet-mediated activation of the CaMKK*β*-LKB1-AMPK pathway attenuates diabetic nephropathy in db/db mice by modulation of apoptosis and autophagy. *Cell Death & Disease*.

[24] Cheng S., So W., Zhang D., Cheng Q., Boucher B., Leung P. (2016). Calcitriol reduces hepatic triglyceride accumulation and glucose output through Ca2+/CaMKK*β*/AMPK activation under insulin-resistant conditions in type 2 diabetes mellitus. *Current Molecular Medicine*.

